# Etching-Assisted Ablation of the UV-Transparent Fluoropolymer CYTOP Using Various Laser Pulse Widths and Subsequent Microfluidic Applications

**DOI:** 10.3390/mi9120662

**Published:** 2018-12-15

**Authors:** Keisuke Nemoto, Yasutaka Hanada

**Affiliations:** Graduate School of Science and Technology, Hirosaki University, 3 Bunkyo-cho, Hirosaki, Aomori 0368561, Japan; h16ms625@gmail.com

**Keywords:** nanosecond laser, ablation, etching, microfabrication, fluoropolymer, CYTOP, microfluidics

## Abstract

This work demonstrated the surface microfabrication of the UV-transparent fluoropolymer CYTOP (perfluoro 1-butenyl vinyl ether), by etching-assisted ablation using lasers with different pulse widths. In previous studies, we developed a technique for CYTOP microfluidic fabrication using laser ablation followed by etching and annealing. However, this technique was not suitable for some industrial applications due to the requirement for prolonged etching of the irradiated areas. The present work developed a faster etching-assisted ablation method in which the laser ablation of CYTOP took place in fluorinated etching solvent and investigated into the fabrication mechanism of ablated craters obtained from various pulse width lasers. The mechanism study revealed that the efficient CYTOP microfabrication can be achieved with a longer pulse width laser using this technique. Therefore, the rapid, high-quality surface microfabrication of CYTOP was demonstrated using a conventional nanosecond laser. Additionally, Microfluidic systems were produced on a CYTOP substrate via the new etching-assisted laser ablation process followed by annealing within 1 h, which is faster than the prior work of the microfluidic chip fabrication. Subsequently, CYTOP and polydimethylsiloxane substrates were bonded to create a 3D microfluidic chip that allowed for a clear microscopic image of the fluid boundary.

## 1. Introduction

Fluoropolymers, as typified by polytetrafluoroethylene (PTFE), have become widely used in electrical [1,2], medical [3,4] and biological [5,6,7] fields due to their unique characteristics such as chemical stability, an electrically-insulating nature and flexibility [8]. The amorphous fluoropolymer CYTOP (perfluoro 1-butenyl vinyl ether) also possesses these same properties in addition to having a high degree of transparency from the deep ultraviolet (UV) to infrared radiation (IR) wavelength regions and almost the same refractive index as water [9]. The high transparency similar to that of fused silica, which is used in biochips, prevents autofluorescence and a low refractive index can allow clear microscopic images to be obtained at fluid boundaries [10]. Therefore, CYTOP is a promising biochip material. With respect to polymer microfabrication, a number of studies using conventional nanosecond (ns) lasers have been reported since the pioneer work of polyethylene terephthalate photoetching by Srinivasan et. al., [11]. Nowadays, excimer and CO_2_ lasers are common tools for prototyping microfluidic devices made of a variety of polymers [12,13,14,15,16]. The laser microfabrication, referred to as laser ablation of polymers, is usually a combination of photochemical and photothermal processes [17]. However, precisely because of their high degree of transparency and significant chemical stability, most fluoropolymers (including CYTOP) are difficult to use as raw materials for microfabrication unless high-energy photon beams such as extreme ultraviolet (XUV) or F_2_ lasers are used as the light sources [18,19].

In the meanwhile, our group developed a technique for the three-dimensional (3D) microfluidic fabrication of a CYTOP substrate using a femtosecond (fs) laser and demonstrated the microscopic observations of a moving cell at a fluid sidewall that were clearer than those obtained using a conventional glass biochip in prior work [10]. Additionally, we have established a method for the high-quality surface microfabrication of CYTOP using a conventional picosecond (ps) green laser for industrial applications [20]. However, during such prior work, it was found that etching using a diluted fluorinated solvent requires a significant amount of time (6 h in the case of Ref. [20]) to remove carbonized residues deposited in the laser-ablated regions, since the photothermal process is dominant in the case of the green laser ablation of UV-transparent CYTOP. In order to shorten the processing time, we attempted to develop a process for the etching-assisted ablation of a CYTOP substrate and investigated the fabrication mechanism associated with lasers having different pulse widths. Following the development of this fabrication technique, we demonstrated 3D microfluidic fabrication by bonding CYTOP and polydimethylsiloxane (PDMS) substrates, and confirmed that clear microscopic images could be obtained at the resulting fluid boundaries.

## 2. Materials and Methods

**Figure 1:**
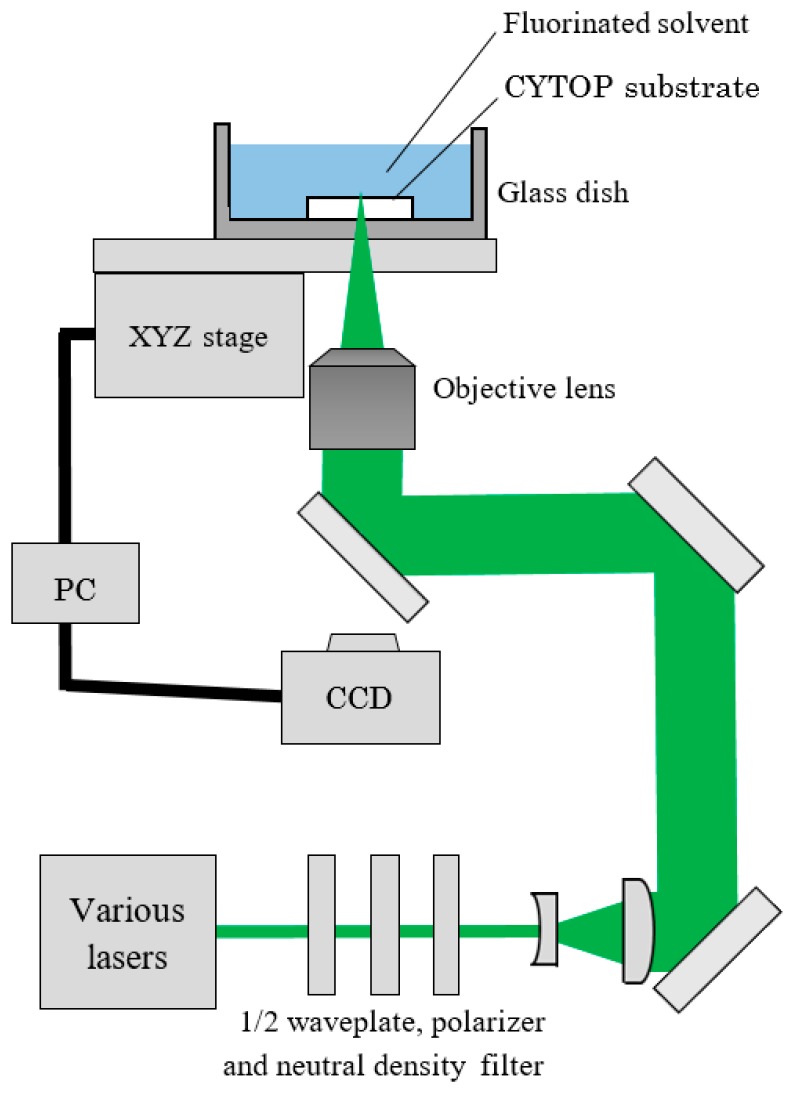
A schematic illustration of the experimental setup.

In the previous studies of CYTOP microfabrication, namely, the laser ablation of CYTOP substrate followed by successive etching using fluorinated solvent, prolonged etching time of the ablated areas was required. Therefore, we attempted the etching-assisted ablation of CYTOP as shown in a schematic diagram of the experimental setup in Figure 1. The commercially-available, UV-transparent fluoropolymer CYTOP (CTX-809SP2, Asahi Glass Co., Ltd., Tokyo, Japan) was used as the substrate. The light sources consisted of a ps laser (Nd:YAG/Cr:YAG laser: 532 nm, 500 ps, 1 kHz), ns laser (Nd:YAG laser: 532 nm, 5 ns, 15 Hz) and fs laser (Ti:sapphire laser: 775 nm, 180 fs, 1 kHz). Each laser pulse energy was adjusted with neutral density filters, a half-wave plate and a polarizer, and were held constant at the energy for stable laser ablation in all the experiments (7.0, 70.0 and 0.4 μJ/pulse for ps, ns and fs laser experiments, respectively). The laser beam was focused at the interface between the CYTOP surface and the etching solvent by an objective lens (×20, numerical aperture = 0.46), with the laser beam approaching from underneath the CYTOP substrate. The fluorinated solvent AC-6000 (Asahi Glass Co., Ltd., Tokyo, Japan) diluted 1:1 by volume with acetone was used as the etching solvent. The glass dish containing the CYTOP substrate and the fluorinated solvent was moved using a PC-controlled x-y-z stage for laser scanning. Every experiment was repeated five times and the mean value of the raw data was adopted. Following the fabrication process, the surface characteristics of the unit were analyzed by transmitted light microscopy (TLM), laser scanning microscopy (LSM), scanning electron microscopy (SEM), and atomic force microscopy (AFM).

Subsequently, microfluidics were fabricated on the CYTOP substrate using the ns laser, maintaining the laser energy at 70 μJ and the laser scanning speed at 60 μm/s. Following the microfluidic fabrication, PDMS substrate (SYLGARD 184, Dow Corning Toray, Tokyo, Japan) was bonded to the fabricated CYTOP microfluidics for creation of 3D microfluidics.

## 3. Results and Discussion

### 3.1. Etching-Assisted Ablation vs. Ablation Followed by Etching

To investigate the etching-assisted ablation of the CYTOP substrate, we compared the craters fabricated by etching-assisted ablation and by laser ablation followed by 1 h etching in an ultrasonic bath, using different pulse width lasers. In the case of the etching-assisted ablation process, the CYTOP substrate was removed from the etching solvent immediately after the laser irradiation, so that only the effects of the laser ablation and etching interaction would be investigated.

**Figure 2:**
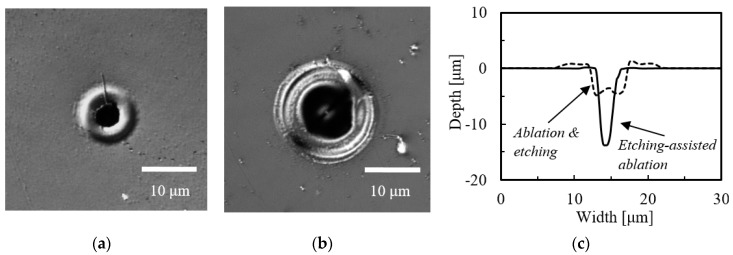
LSM images of craters fabricated by (**a**) etching-assisted ablation and (**b**) ablation followed by 1 h etching (both using a ps laser); (**c**) a cross-sectional view of the craters in (**a**,**b**).

Figure 2a,b shows LSM images of the craters fabricated on the CYTOP surface by etching-assisted ablation using a ps laser and by ps laser ablation followed by 1 h etching in an ultrasonic bath. In both experiments, the substrate was ablated using only a single laser pulse at a laser energy of 7.0 μJ. Figure 2c presents a cross-sectional view of the craters shown in the LSM images. It is evident that the melted region around the crater made by etching-assisted ablation is smaller compared with that fabricated by ablation followed by etching. In addition, the former crater was deeper but narrower, and thus had a higher aspect ratio of 3.34. This confirms the improved resolution of the microfabrication process.

**Figure 3:**
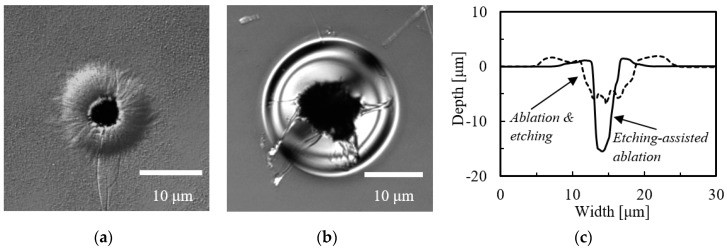
LSM images of craters fabricated by (**a**) etching-assisted ablation and (**b**) ablation followed by 1 h etching (both using a ns laser); (**c**) a cross-sectional view of the craters in (**a**,**b**).

We also performed the same experiment using the ns laser at the laser energy of 70.0 μJ/pulse. Figure 3a,b shows LSM images of the resulting craters, while (c) provides cross-sections of these craters. Again, the etching-assisted ablation generated a crater with a higher aspect ratio of 3.52. In addition, the increased ratio of crater depth obtained from the cases of etching-assisted ablation and ablation followed by etching was larger compared to that obtained from the case of the ps laser (in case of ns laser: 2.9, ps laser: 2.6 obtained from Table 1, described later), even though the laser energy was respectively kept constant in each laser experiment.

**Figure 4:**
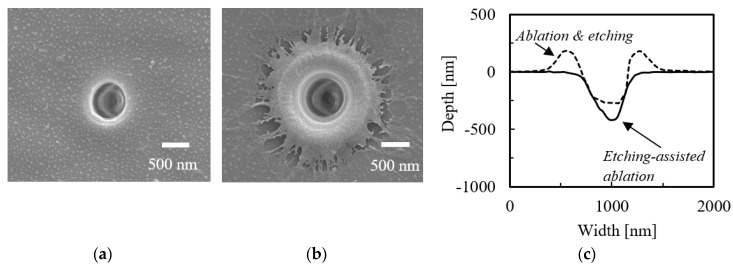
SEM images of craters fabricated by (**a**) etching-assisted ablation and (**b**) ablation followed by 1 h etching (both using a fs laser); (**c**) a cross-sectional view of the craters in (**a**,**b**) obtained from AFM measurements.

Figure 4 demonstrates that applying the fs laser at the laser energy of 0.4 μJ/pulse during the etching-assisted ablation did not produce a melted region around the crater, and that the crater diameter was ca. 700 nm. The increased ratio of crater depth was 1.6, namely, the smallest increase ratio was obtained using the fs laser compared to those obtained from the ps and ns lasers written above.

Table 1 summarizes the diameters, depths and aspect ratios of the craters; these results confirm that the aspect ratios of the craters were significantly improved when etching-assisted ablation was employed at all pulse widths. In addition, the aspect ratios of the etching-assisted ablation craters increased as the pulse width was increased, with especially deep etching in the case of the ns laser.

**Figure 5:**
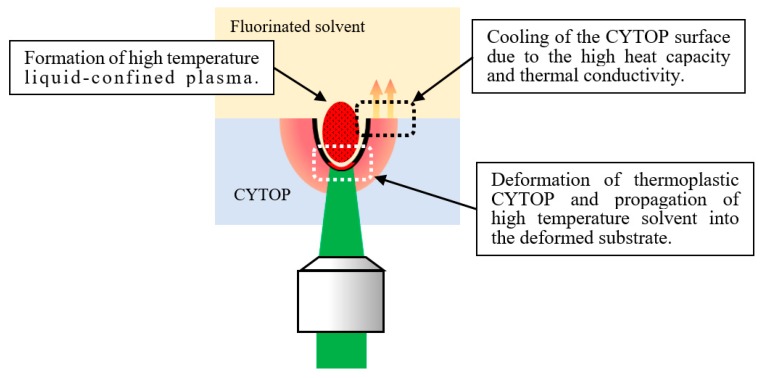
A schematic illustration of the etching-assisted ablation mechanism.

Based on these data, we provide a proposed mechanism for the etching-assisted ablation in conjunction with a longer pulse width in Figure 5. In this process, first, liquid-confined laser-induced plasma with high temperature is generated by the laser ablation at the fluorinated solvent–CYTOP boundary. At this stage, the solubility of the polymer is enhanced since CYTOP is thermoplastic and the etching process is strongly dependent on the temperature of the solvent. Thus, following the laser irradiation, high-temperature etching solvent propagates into the deformed laser-irradiated area, resulting in more efficient etching of the CYTOP substrate. The use of a longer pulse width laser simply increases the interaction time between the CYTOP and the etching solvent, also resulting in effective CYTOP etching. Furthermore, the molten region around the crater obtained by the etching-assisted ablation is decreased. This is because the etching solvent prevents heat accumulation by absorbing the extra heat from the laser-induced plasma due to its heat capacity and thermal conductivity (with respect to air in the case of laser ablation followed by etching). This results in an efficient cooling of the polymer. Moreover, unlike in air, most of the etching-assisted ablated CYTOP polymer does not redeposit back onto the substrate but dissolves into the fluorinated etching solvent. Therefore, the longer pulse width laser, in our case, the ns laser would be the appropriate light source for the efficient microfabrication of CYTOP substrate using etching-assisted ablation.

### 3.2. Microfluidic Fabrication Using Etching-Assisted ns Laser Ablation

**Figure 6:**
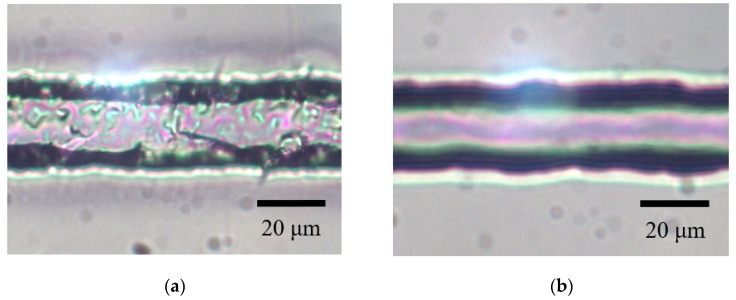
TLM images of the microfluidic structure fabricated on the CYTOP surface after (**a**) multiple laser scanning by etching-assisted ablation and (**b**) subsequent annealing.

Following the development of this surface microfabrication technique, etching-assisted ablation using the ns laser was employed to create microfluidics on a CYTOP surface. Multiple laser scans were initially performed so as to fabricate microfluidics over the desired regions. Figure 6 presents TLM images of the specimen after multiple laser scans and the subsequent annealing of the substrate. These trials were performed using a laser energy and scanning speed of 70 μJ/pulse and 60 μm/s, respectively. When fabricating patterns over large areas of the CYTOP substrate, the laser beam was scanned line by line over the x-y plane with a pitch of 4 μm so as to fabricate the desired region. Based on results from prior work, the sample was annealed at 160 °C for 30 min after etching to smooth the surface [20]. Figure 6 demonstrates that we were able to produce a microfluidic structure with a width of ca. 20 μm and a depth of ca. 15 μm on the surface of the CYTOP substrate. Additionally, the rough, degraded surface evident on the bottom of the microfluidics in Figure 6a was smoothed as a result of the annealing.

**Figure 7:**
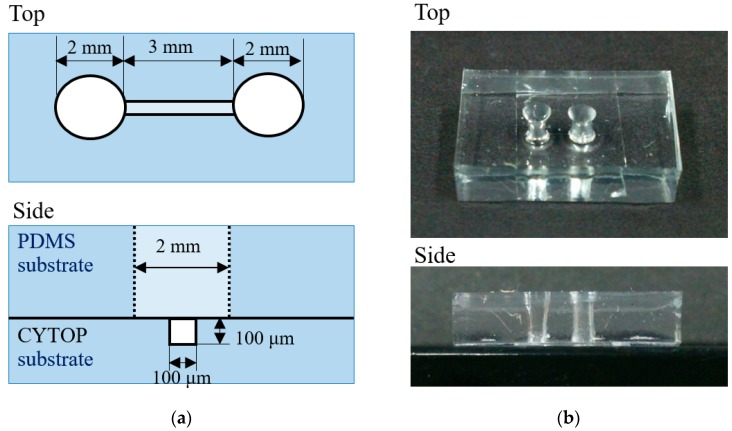
(**a**) A schematic illustration and (**b**) photographic images of the fabricated microfluidic chip.

**Figure 8:**
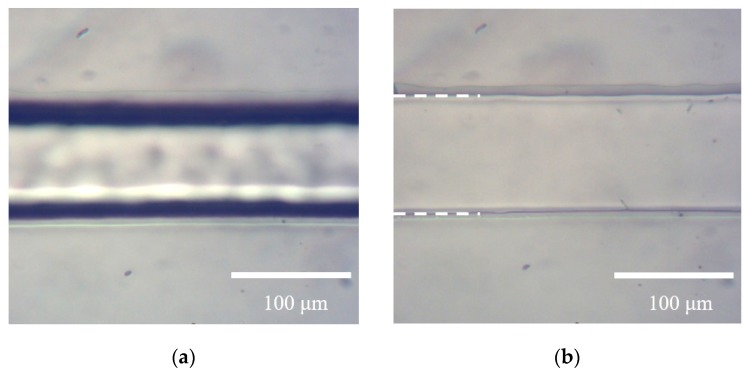
TLM images of the microfluidics (**a**) before and (**b**) after filling with water.

Following this trial confirming the successful etching-assisted ablation of a large area, a 3D microfluidic chip was fabricated by bonding CYTOP and PDMS substrates. Figure 7 shows schematic diagrams and photographic images of the resulting chip. This unit incorporated two open reservoirs that were mechanically punched through the PDMS substrate as well as microfluidics fabricated by the etching-assisted ablation of the CYTOP substrate. During the formation of microfluidics, multiple laser scans were performed in the x-y plane while shifting the laser beam 10 times along the z-direction with a pitch of 10 μm, giving a pattern with a 100 μm depth. Following the fabrication of CYTOP and PDMS substrates, two workpieces were simply bonded by stacking the substrates due to the strong adhesiveness of PDMS. By doing so, a 3D microfluidic chip was fabricated within 1 h which is faster than the prior work of the microfluidic chip fabrication [10,20]. Figure 8 provides microscopy images obtained from the base of the microfluidics immediately after fabrication as well as after filling with water from one of the reservoirs. It is very difficult to distinguish the fluid interface when the microfluidic pattern is filled with water due to the minimal refractive index mismatch between the fluid sidewall and the water. This result demonstrates that the newly-developed etching-assisted ablation technique using a ns laser permits the high-quality, high-speed production of a microfluidic chip, representing an alternative approach to studying solid boundaries such as cell-fluid interfaces.

## 4. Conclusions

This work examined the etching-assisted ablation of the UV-transparent polymer CYTOP and also fabricated the 3D microfluidic chip to obtain clear microscopic images at the fluid sidewall. The fundamental characteristics of the etching-assisted ablation using various pulse width lasers were investigated and the longest pulse width was found to be optimal. Using this technique, craters with smaller diameters can be obtained due to the efficient cooling at the interface between the CYTOP surface and the etching solvent. In addition, the solubility of the CYTOP is enhanced as a result of increased temperature at the laser-irradiated area, thus generating deeper craters with high aspect ratios. A CYTOP microfluidic chip was created to demonstrate clear microscopic images at the fluid interface. Etching-assisted ablation using a conventional ns laser was confirmed to be an efficient approach to the surface microfabrication of CYTOP, and this technique may, therefore, have applications in the study of cell-surface interactions.

## Figures and Tables

**Table 1:** Diameters, depths and aspect ratios of fabricated craters.

Laser & Method		Diameter [μm]	Depth [μm]	Aspect Ratio
ns laser	Ablation & etching	7.5 ± 0.03	5.4 ± 0.02	0.72
	Etching assisted ablation	4.4 ± 0.05	15.5 ± 0.02	3.52
ps laser	Ablation & etching	5.7 ± 0.05	5.2 ± 0.01	0.91
	Etching assisted ablation	4.1 ± 0.02	13.7 ± 0.05	3.34
fs laser	Ablation & etching	0.70 ± 0.02	0.27 ± 0.03	0.39
	Etching assisted ablation	0.71 ± 0.03	0.42 ± 0.01	0.59
